# Indoleamine-2,3-Dioxygenase as a Perioperative Marker of the Immune System

**DOI:** 10.3389/fphys.2021.766511

**Published:** 2021-11-08

**Authors:** Corina Bello, Paul Philipp Heinisch, Maks Mihalj, Thierry Carrel, Markus M. Luedi

**Affiliations:** ^1^Department of Anaesthesiology, Spital Grabs, Grabs, Switzerland; ^2^Department of Anaesthesiology and Pain Medicine, Bern University Hospital (Inselspital), University of Bern, Bern, Switzerland; ^3^Department of Congenital and Pediatric Heart Surgery, German Heart Center Munich, Technical University, Munich, Germany; ^4^Department of Cardiovascular Surgery, Bern University Hospital (Inselspital), Bern, Switzerland; ^5^Department of Cardiovascular Surgery, University Hospital Zurich, Zurich, Switzerland

**Keywords:** immune system, cardiac surgery, indoleamine-2, 3-dioxygenase, tryptophan, immunosuppression

## Abstract

Indoleamine-2,3-dioxygenase (IDO) is the “rate-limiting” enzyme in the kynurenine (Kyn) pathway of the tryptophan (Trp) catabolism. By its immune-modulatory effect, IDO initiates changes to the physiologically balanced immune state and plays a key role in the pathogenesis of various diseases, as well as in the perioperative setting during surgery. In autoimmune processes, highly malignant cancers such as glioblastoma or organ transplantation, IDO’s involvement has been studied extensively. However, in severe systemic infections, as present in sepsis, it is not yet completely understood. Hereafter, in this narrative review, we present the current knowledge of IDO’s implication on such complex immune-related processes. Moreover, we address the role of IDO as a predictive biomarker as well as a therapeutic target for immune-mediated diseases. Finally, we discuss IDO in the setting of surgical trauma-induced stress and highlight its promising use as a biomarker in the pre-operative setting for all disciplines involved in the decision-making process and treatment of patients undergoing surgery.

## The Physiological Role of Indoleamine-2,3-Dioxygenase

Physiologically, homeostasis in the immune system is always maintained through a balance between protection from infections versus prevention of excessive autoimmune reactions. Cellular immune response including B- and T-lymphocytes has a major role in the maintenance of an immunological equilibrium within the individual immune system. In addition, they play an important role in self-tolerance (Abbas and Pillai, 2019).

The differentiation of T cells into different subtypes with diverse activities depends greatly on so-called fate-specific cytokines and receptors (Abbas and Pillai, 2019). Nowadays, it is thought that the differentiation of cluster of differentiation (CD-)4 naive cells into T regulatory cells (Tregs) is caused by a combination of T-cell receptor activation, CD-28 signaling, FOX-P3 stimulation (Fontenot et al., 2003) *via* interleukin (IL-)2, and many other cytokines (IL-15 and IL-7), as well as individual interactions by themselves (not *via* FOXP3), including NFAT/AP1, ICOS/ICOSL, and thymic stromal lymphopoietin (Watanabe et al., 2005; Mantel et al., 2006; Nazzal et al., 2014).

T regulatory cells then interact with other T cells, natural killer cells, the host cell membrane, B cells, and monocytes. Some important anti-inflammatory mediators mitigating these effects include transforming growth factor beta (TGF-β) IL-2, interaction of programmed death-ligand 1 (PD-L1) and programmed cell death protein 1 (PD-1) (with B cells), cytotoxic T-lymphocyte-associated protein (CTLA-4), and lymphocyte-activation gene 3 (LAG3). CTLA-4 has important downstream effects on dendritic cells (DCs): it decreases antigen-capturing and antigen-presenting capability and induces indoleamine-2,3-dioxygenase (IDO) (Schmidt et al., 2012).

Indoleamine-2,3-dioxygenase catalyzes the rate-limiting step in the Kyn pathway, catabolizing the essential amino acid tryptophan (Trp). Trp catabolism’s Kyn arm is dependent on IDO, which exists in two isoforms: IDO1 and IDO2. IDO2 has a modest enzymatic potential (Pantouris et al., 2014) and has received less attention. Tryptophan-2,3-dioxygenase is the third enzyme engaged in the Kyn pathway. As a result, our review will focus exclusively on the role of IDO1.

**FIGURE 1:**
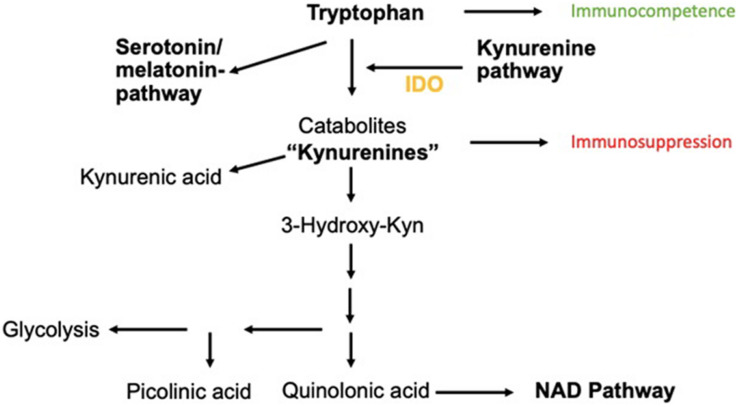
Catabolism of Trp *via* Kyn-pathway. The neuroinflammatory metabolites kynurenic acid, picolinic acid, and quinolinic acids are shown as well as the transition to the NAD pathway important for cellular energy generation.

This enzyme catalyzes the reaction of Trp to *N*-formylkynurenine, leading to the production of Kyn metabolites. Kyn metabolites are broken down into metabolites such as picolinic acid, quinolinic acid, and kynurenic acid. These metabolites are actively involved in neuroinflammatory diseases and also serve as a substrate for the generation of nicotinamide adenine dinucleotide (NAD), a key molecule for cellular repair, energy substrate, and fatigue (Figure 1).

Cell-mediated immune response is massively regulated by IDO depending on immunosuppressive effects. It is a potentially ground-breaking pathway that could serve as a target for therapy of many human immune-mediated diseases, ranging from autoimmune disease (Anquetil et al., 2018) to chronic infections. In addition, it may serve as a marker of immune status, e.g., in the perioperative setting.

## Which Tissues Express Indoleamine-2,3-Dioxygenase?

Indoleamine-2,3-deoxygenase is expressed and normally active in many tissues, such as placental endothelial cells, parenchymal lung cells, cells of the epithelial lining of the female genital tract, and most relevant for this review, DCs (Théate et al., 2015). There is extensive interest in the inducibility of IDO1. While not constitutively expressed, its transcription can be activated or enhanced under certain conditions; for example, in and around tumor cells or in antigen-presenting cells. In addition, endothelial cells, innate immune cells, mesenchymal cells, lymph nodes draining tumor tissue (Prendergast et al., 2018), and mononuclear blood cells (Meireson et al., 2020) can express IDO. In addition, the expression of IDO increases in elderly people (Marttila et al., 2011).

The mechanisms by which the enhancement of this crucial enzyme is mediated are not completely understood. There are numerous regulatory and modulatory coenzymes involved in the Trp metabolism, influencing a balanced state between serotonin and Kyn. One of these is Coenzyme Q (Abuelezz et al., 2017). Also, stress can induce the Kyn pathway, while exercise can lead to a decrease in stress-induced high Kyn levels (Agudelo et al., 2014; Kirmeier et al., 2019). Flavonoids such as curcumin deactivate IDO interaction with the aryl hydrocarbon receptor (AhR) (Busbee et al., 2013). Through modulation of the AhR, such substances can alter macrophage IDO activity and block interferon (IFN-)gamma-induced IDO activity (Jeong et al., 2009). Although not the main promoting factor for IDO expression (Taylor and Feng, 1991), IFN-y plays an immunomodulatory role by affecting response to viral and bacterial infections, promoting “leukocyte trafficking,” and boosting the processing of antigens and apoptosis of infected cells.

**FIGURE 2:**
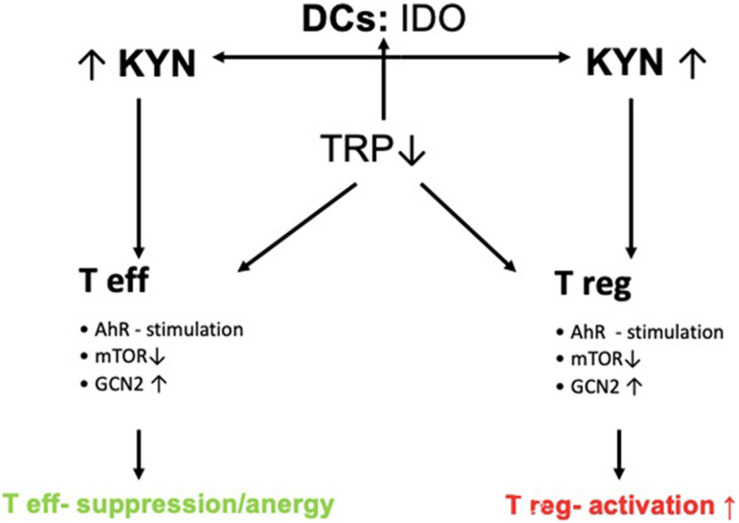
Control of T-cell response by IDO. DCs express IDO thereby leading to increased Kyn-metabolite production. Increased Kyn-metabolites stimulate AhR and GCN2 and inhibit mTOR thereby leading to the activation of Tregs and inducing tolerance *via* suppression of effector T-cells (Teffs).

Effector T-cells produce IFN-y in infective states. Together with pattern recognition receptors (PRRs) and cytokines such as tumor necrosis factor (TNF-)alpha, IL-4 lipopolysaccharide, and Type-I interferons, IFN-y then acts as a key initiator of the pro-inflammatory cascade (Kak et al., 2018) (Figure 2).

**FIGURE 3:**
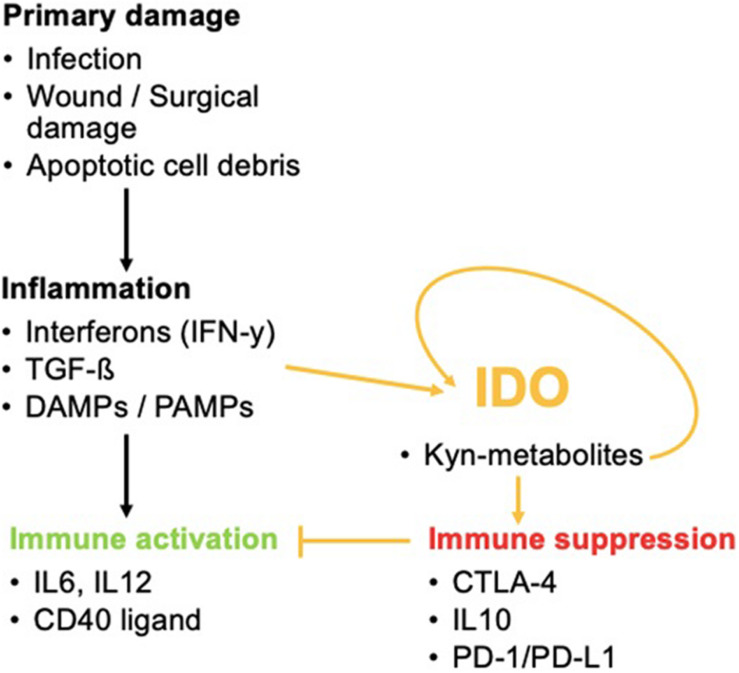
The role of IDO in inflammatory processes. IDO activation by inflammation favors immune tolerance and hinders immune activation that would normally occur *via* the activation of pro-inflammatory cytokines. IDO can even promote its own activity once activated.

In addition, it is a potent initiator of the Kyn pathway. Other IDO-elevating factors include IL-10, IL-27, the cytokine production of Treg cells including TGF-β, IL-10, and adenosine or Treg-expression profile of CTLA-4. These lead to enhanced IDO1 secretion by DCs as well as cyclooxygenase (COX-)2 and prostaglandin E2 (PGE2). COX-2 and PGE2 are associated with IDO1 expression in tumor cells (Hornyák et al., 2018). Once the balance of Trp metabolism is shifted to the Kyn pathway, Kyn products induce a self-enforcing feedback loop involving IDO expression. This paves the way for a vicious cycle during inflammation (Litzenburger et al., 2014) (Figure 3).

## Pathways by Which Immune Regulatory Function Is Mitigated

Once activated, there are three main pathways by which the immune regulatory function of IDO is mitigated. First, Trp catabolites produced by IDO bind to the AhR. This receptor is a cytosolic transcription factor. *Via* IL-6-induced signal transducer and activator of transcription protein (STAT-)3 signaling, the differentiation of Treg and DCs is promoted and apoptosis of T helper (T_*h*_)1 cells is induced. Second, the Kyn pathway is stimulated, activating a positive feedback loop until depletion of this amino acid. Immune cells lacking Trp are suppressed, while tolerogenic immune cells are activated (Zhai et al., 2018). In detail, general control non-depressible 2 (GCN2) kinase and mechanistic target of rapamycin (mTOR, previously referred to as the mammalian target of rapamycin) sense low tryptophan levels and promote anergy as well as differentiation into regulatory types in effector T-cells (Teffs) (Munn and Mellor, 2013). Also, T_*h*_17 diffferentiation is altered (Albini et al., 2017). The third pathway of immune modulation that has been identified is executed *via* nuclear factor kappa-light-chain-enhancer of activated B cells (NF-κB). IDO may act as a signaling protein inducing NF-κB, which then joins TGF-β in favoring the survival of regulatory plasmacytoid DCs. These cells suppress T-cell activity and promote Treg formation, while keeping the production of pro-inflammatory cytokines at a low level. The effect of IDO might not be limited to plasmacytoid DCs, but rather lead *via* non-enzymic function to an upregulation of Src homology 2 domain phosphatase (SHP-)1 and SHP-2 receptors on DCs. This in turn favors binding of SHP1/2 to immune tyrosine-based inhibitory motif (ITIM)1 over ITIM2. In this way, a cascade is finally activated, leading to long-term expression of the IDO gene in the nucleus of DCs of the plasmacytoid and conventional type (Pallotta et al., 2011).

Lately, IDO has been found to have a direct anti-infective effect by suppressing the replication of certain bacteria and parasites as well as preventing viral spread. Trp depletion also seems to have a bactericidal effect, as found in infections with toxoplasma, or to serve as a virostatic agent through depletion of IFN-y in people infected with cytomegalovirus. This effect could be reversed by the administration of exogenous tryptophan. Altering such regulatory effects of IDO may also help certain pathogens infect human cells without being noticed by the immune system. Uropathogenic *Escherichia coli* induce IDO in epithelial cells, thereby successfully colonizing urinary epithelium (Schmidt and Schultze, 2014).

The role of IDO is complex, and there are many ongoing studies looking into the effects of various signaling proteins involved in the immune response to IDO. The extent of IFN-y-induced activation of IDO, for example, can be altered by B lymphocyte-induced maturation protein-1 (BLIMP-1) binding to the IFN-y binding site on IDO (Barnes et al., 2009). BLIMP-1 itself is promoted by GCN2, and thereby plays a key role in regulating IDO activation in response to GCN2-sensed Trp-depletion. mTOR-dependent IDO activation *via* response to Trp depletion, on the other hand, can be decreased by 1-methyl-DL-tryptophan (1-MT). The D isoform acts as a Trp-mimetic at mTOR (Moon et al., 2015).

In view of the fact that the role of IDO is already very complex in physiological states, and given that IDO expression is altered under the circumstances discussed above, the role of IDO in modulating the course of immune-mediated disease is of great interest to many specialties.

## Autoimmunity and Neurodegenerative Disorders

Indoleamine-2,3-dioxygenase has been identified as a driving factor in certain human diseases in which the equilibrium between immune response and self-tolerance is disrupted, such as in some autoimmune diseases. For example, altered IDO levels have been found in patients suffering from Type 1 diabetes, with IDO1 being physiologically highly expressed in pancreatic islets β-cells of healthy tissue, but absent in the remainder (Anquetil et al., 2018). Also, in Type 2 diabetes, IDO levels are excessive (Baban et al., 2013). Elevated IDO levels have also been observed in autoimmune neurodegenerative conditions such as multiple sclerosis (MS), in neuropsychiatric diseases such as depression, and in inflammatory bowel disorders such as Crohn’s disease and systemic sclerosis. This may indicate a possible role in the pathogenesis of these conditions (Pallotta et al., 2011).

## Organ Transplantation

Autoimmunity or tolerance in a broader sense is also a key factor following organ transplantations. In acute liver transplant rejection, the severity of acute organ rejection amounts to IDO-positive cells in the portal area, where IDO positivity in liver cells was associated with worse host tolerance of a transplanted organ (Sun et al., 2012).

On the contrary, high levels of Trp catabolism were associated with better long-term survival in kidney transplant recipients (Brandacher et al., 2007) or small bowel transplantations. This has also been investigated when IDO activity was externally induced in the recipients by pharmacological measures such as transfusion of IDO-positive DC to hosts of small bowel transplants (Xie et al., 2015). IDO might therefore be a potent target for further improvements in the long-term success of transplantations, once its role in autoimmune balance is better understood.

## Glioblastoma

Currently, research on IDO is most advanced in cancer cells, which quite commonly express IDO (Théate et al., 2015). Glioblastoma and its surrounding cells do not normally express IDO; however, this might contribute to the immunocompromised microenvironment of malignant gliomas (Théate et al., 2015).

Aging seems to further decrease IDO expression, making glioblastoma patients at an advanced age less likely to respond well to immunotherapy due to IDO’s immunosuppressive effect (Ladomersky et al., 2018). However, treatment with the pro-inflammatory factor IFN-y can induce IDO in such cancer cells (Zhai et al., 2017).

Promoting IDO is a promising method for targeting glioblastoma, which is highly resistant to most of the commonly used cancer treatments and often requires a combination of surgical resection, chemotherapy, radiotherapy, and/or immunotherapy (Minniti et al., 2009). Directly targeting IDO for therapeutic use is promising, but has mostly been used for cancer treatment so far. Immunotherapy can target the Trp metabolism. In Trp-depleted cancer cells, protein synthesis and thereby proliferation potential is decreased through the activation of an intracellular stress response *via* GCN-2. CD8-T-cell survival is promoted through GCN-2 activity in Trp-depleted states (Rashidi et al., 2020). However, *in vivo* Trp levels show very low fluctuations in the brain by an autoregulatory mechanism (Adam et al., 2018). Also, some evidence indicates negative effects of low Trp levels changing HLA-1-molecule presentation of melanoma cells rendering them immunity against the immune system of the host (Bartok et al., 2021). The role of IDO in NAD generation in glioma cells has also been studied extensively, and it is supposed to be highly involved in the development of resistance to therapy (Rosell et al., 2011). Additionally, changes in AhR and AhR target gene levels have been linked to increased IDO1 and TDO2 levels in glioblastoma cells pointing to the fact that increased IDO-activity might promote AhR-mediated changes in glioma cells that enable immune escape and malignant potential (Opitz et al., 2011; Sadik et al., 2020). AhR plays an important role in clonogenic survival (Opitz et al., 2011; Sadik et al., 2020), motility (Opitz et al., 2011; Sadik et al., 2020), promoting pro-oncogenic cytokine production such as TGF-β (Gramatzki et al., 2009), immunosuppression by its effects on T-cells (Ehrlich et al., 2018; Prasad Singh et al., 2020), DCs (Goudot et al., 2017), macrophages (Goudot et al., 2017), natural killer cells (Shin et al., 2013), B-cells (Piper et al., 2019), or glioblastoma-specific tumor-associated macrophages (Takenaka et al., 2019). Finally, AhR and AhR genes induce molecular immune checkpoints such as PD-L1 (Liu et al., 2018).

The mechanisms by which IDO enhancement may help fight glioblastoma cells are still not clear, and agents acting solely on IDO without simultaneously targeting other immune checkpoints such as PD-L1 are not effective (Ladomersky et al., 2018). Whether or not the higher efficacy when IDO-enhancement and PD-L1 blockade are applied jointly might be based on IDOs promoting effect on PD-L1 expression remains unclear. In patients at an advanced age, combining different therapeutic approaches including IDO is less effective than in younger glioblastoma patients (Bulloch et al., 2008). More studies are needed to further develop treatment plans, especially for highly malignant, immune-escaping, and therapy-resistant cancer types such as glioblastoma.

## Indoleamine-2,3-Dioxygenase as a Predictive Biomarker

Aside from the therapeutic potential of IDO and its metabolites, it may be a promising biomarker with predictive value in cancer. Predictive biomarkers have a substantial significance because they may allow more accurate expectations for the treatment teams and the patients. These should aid in the selection of the best possible treatment regimen.

Indoleamine-2,3-dioxygenase activity has recently been analyzed with regard to progression of lung cancer. IDO2 was found to have prognostic and predictive value together with PD-L1-expression for NSCLC (Mandarano et al., 2020).

Indoleamine-2,3-dioxygenase 1 levels could be of interest for perioperative decision-making, with some studies suggesting its usefulness in deciding on perioperative immunotherapy directly targeting certain immune checkpoints (Takada et al., 2020). Analysis of IDO level was even effective for decision-making on usefulness of perioperative chemotherapy in early-stage adenocarcinoma of the lung (Takada et al., 2020). Thus IDO provides both therapeutic and diagnostic value in the field of oncology.

## Infections

Infections also cause an inflammatory condition that promotes the growth of cancer cells over time. Infections can be chronic or acute, and the Kyn pathway is involved in the development of both. Immune-mediated pathways are inadequate in combating an infective agent in persistent infections. An inflammatory condition is maintained or improperly prolonged.

The role of IDO in infection is to suppress the replication of intracellular pathogens and to create an immune balance by regulating T-cell differentiation (Mbongue et al., 2015). In chronic infections such as hepatitis C, inflammatory mediators such as IFN-β or IFN-y induce IDO expression (Schulz et al., 2015; Yoshio et al., 2016). Hepatitis B replication, on the other hand, can be suppressed by IFN-y *via* Trp depletion (through activation of IDO), helping infected cells mount anti-viral responses (Mao et al., 2011). Some pathogens use this immunoregulatory response to their advantage and inhibit IDO activity allowing an escape of immunological checkpoints and persist and mature in host cells (Planes and Bahraoui, 2013). Epstein–Barr virus is such a virus that escapes the immune response by interacting with IDO (Liu et al., 2014).

Another more commonly seen problem, especially after surgery, are surgical site infections. Lately, effects of Kyn metabolites have been identified as key drivers in dysregulation of wound healing (Kim et al., 2021), but more profound analyses are needed.

## Influenza

Apart from the important role of IDO in chronic infective conditions, the Kyn pathway also contributes to an understanding of acute infections. Lung inflammation induced by influenza might be highly dependent on IDO activity. IDO with its immunosuppressive effect might predispose infected lung tissue to bacterial infection. By inhibiting IDO activity, heterosubtypic T memory cells are favorably activated. These cells then provide cross-protection immunity against influenza virus (Sage et al., 2014).

In view of these findings, the response to yearly influenza vaccines could probably be improved by inhibiting IDO. The promoted activation of T memory cells by IDO inhibition may improve individual immunity against different influenza subtypes.

On the other hand, enhancing IDO could give the immune response support to fight infections with parainfluenza virus. IDO depletes parainfluenza virus of L-Trp and thereby inhibits its metabolism (not by other metabolites of the Kyn pathway, though). Interestingly, 5-hydroxy-tryptophan, a Trp isoform favoring the serotonin pathway, protects parainfluenza virus from this IDO-mediated block in metabolism (Rabbani and Barik, 2017).

## Sepsis

Sepsis is a devastating acute infection that creates a state of dysregulation in the immune system in response to an infection with systemic involvement (Raeven et al., 2018). It is difficult to identify and even more difficult to treat once septic shock has occurred. Even for highly competent interdisciplinary teams of treating physicians, it sometimes appears to be a “one-way track” leading to septic shock. It is therefore critical to better understand the mechanics of this acute systemic infection in order to identify therapy targets.

So far, little is known about the roles IDO and the Kyn pathway play in the septic shock spiral. However, increased activity of IDO has been registered lately in patients with severe sepsis and septic shock (Tattevin et al., 2010). Further studies are needed to improve outcome of acute infections – especially sepsis – and to provide evidence-based care.

## Surgical Trauma

Indoleamine-2,3-dioxygenase levels are typically elevated during inflammatory reactions, and this makes it a useful biomarker for detecting immune dysregulation. Surgical trauma is a well-known cause of acute stress, as is an acute infection. Because of this, IDO may prove useful as a biomarker to track the recovery process after surgical trauma.

Surgical stress induces endocrine changes as well as cellular tissue damage. Stress hormones such as cortisol, ACTH, and growth hormone regulate the metabolism of glucose, as well as anabolic and catabolic processes, and generally affect homeostasis, with immune cells mitigating an inflammatory response (Desborough, 2000).

Damage to tissue leads to the activation of immune cells that release damage-associated molecular patterns (DAMPs). DAMPs then activate monocytes and neutrophils, which produce pro-inflammatory cytokines. Such cytokines include IL-1, TNF-α, GCS, or TGF-β among others. DAMPs then activate IL-6, which enhances acute-phase proteins such as C-reactive protein (CRP) and macroglobulin (Desborough, 2000) and decreases important mediators of oxidative stress tolerance such as zinc and iron (Sheeran and Hall, 1997).

Pro-inflammatory conditions result as a consequence of this. Patients undergoing cardiac surgery with extracorporeal circuits, which increases the risk of sepsis and infection, are more likely to have additional tissue damage and a persistent severe inflammatory response syndrome (SIRS). More immune cells will be activated as a result of infections with pathogen-associated molecular patterns (PAMPs), which will lead to a recurrence of SIRS and, ultimately, death (Lord et al., 2014).

Not every patient who has general surgery develops SIRS. However, identifying high-risk individuals is still challenging, and even early identification of surgical trauma-induced SIRS that is already “present” is problematic. More research is needed to identify the typical patients which may be at risk.

## Indoleamine-2,3-Dioxygenase as a Target in Infective Diseases Related to Oxidative Stress

Indoleamine-2,3-dioxygenase involvement in the perioperative phase and for the treatment of sepsis is still in its early stages. The pro-inflammatory cytokines involved are the same as those found in other immune-mediated diseases where IDO has been implicated, such as acute infections. It would be revolutionary to specifically target immune-modulating enzymes like IDO without falling into a vicious loop of SIRS after surgery. Aside from the use of antibiotics to limit microbial development, no therapies have been found to have a substantial postoperative effect on SIRS/sepsis regulation (Lord et al., 2014).

However, a recent study involving pediatric patients undergoing cardiopulmonary bypass (CPB) found higher IL-6, CRP, and IDO levels in children who developed SIRS following CPB. The plasma IDO level was the best diagnostic forecast (Wang et al., 2018). The authors even suggested that IDO is more sensitive than IL-6 or CRP to predict SIRS upon surgical correction of congenital heart diseases. Predicting such negative outcomes could be a crucial advance in optimal patient management prior to surgery. It would also have a significant impact on treatment plans in sepsis and post-surgical inflammation (SIRS), where a change is desperately needed.

Indoleamine-2,3-dioxygenase can also be blocked by CD11b + l cells in peritonitis and postoperative sepsis. This is a protective benefit in hosts during sepsis, and was even found to improve mortality (Hoshi et al., 2014). Decreasing IDO activity by applying suppressive cytokines such as GM-CSF decreases catabolic Kyn metabolites in sepsis. This could improve the antibacterial defense (Schefold et al., 2010). Finally, the administration of melatonin, an IDO inhibitor, can directly inhibit the proinflammatory Kyn pathway (Moreno et al., 2018).

Other benefits of inhibiting IDO include positive effects on oxygen- and nitrogen-based toxic metabolites, stimulation of antioxidative mechanisms, limitation of free radical generation *via* more efficient activity of the electron transport chain, increased formation of ATP, and preservation of mitochondrial integrity. This in turn is key for the maintenance of cell functions and survival (Leon et al., 2004). Endothelial cells cultured in laboratory-induced septic environments were found to have altered NF-κB activity, cytokine profiles, mitochondrial membrane potential, and metabolic changes favoring protection against oxidative mitochondrial stress when exposed to structurally similar compounds to IDO (Lowes et al., 2011).

## Indoleamine-2,3-Dioxygenase as a Target in Anoxic States Related to Oxidative Stress

Oxidative stress and mitochondrial damage can be found in many other conditions such as Alzheimer’s, Parkinson’s, epileptogenic disease, or reperfusion injury after ischemia (Leon et al., 2004). The latter relates to the observation of adverse consequences of reperfusing organs that were exposed to acute ischemia, either as a pathologic event such as acute myocardial infarction or as a therapeutic strategy such as producing hypothermia to protect brain tissue following unconscious cardiac arrest. Induced hypothermia raised the chance of developing a serious infection/sepsis (Schefold et al., 2016). This was directly related to IDO overexpression and Trp suppression caused by low body temperature. Given these latest findings, such commonly used therapeutic techniques may need to be reevaluated, since they may even impair patient outcomes.

**FIGURE 4:**
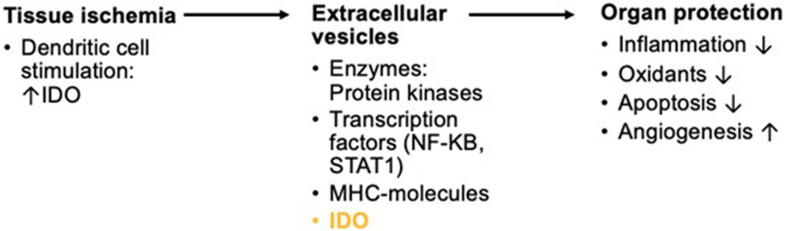
Short overview of the physiologic background in remote ischemic preconditioning for reperfusion of anoxic organs. Ischemia triggers the activation of DCs. Inflammation by tissue ischemia induces IDO in DCs. The production of exosomes with enzymatic composites, transcription factors, major histocompatibility complex (MHC-) molecules, and IDO among many other compounds is triggered. These EVs show protective effects on organs in reperfusion.

Other studies assessing organ damage after induced anoxia such as in renal reperfusion after transplantations have also found an increase in IDO induction. Both hypoxia and reoxygenation of renal tissue led to significant higher IDO levels (Eleftheriadis et al., 2021). Damage caused by reperfusion is to a vast extent due to inflammatory responses (Eleftheriadis et al., 2021). These inflammatory changes might possibly lead to advanced systemic inflammation just an in sepsis or surgical trauma-induced stress. A promising new approach to the prevention of reperfusion injury is the application of extracellular vesicles (EVs) from mesenchymal cells such as DCs. These EVs can also be produced during sheer-stress on the vasculature as part of short periods of ischemia. In so-called remote ischemic preconditioning, ischemia is induced upon a distal part of the body as, for example, the arm by alternatingly inflating and deflating cuffs (Gassanov et al., 2014; Figure 4). The EVs can then be extracted from the distal area and re-introduced into the patient. They express micro-ribonucleic acid (RNA) and seem to have anti-inflammatory, anti-oxidative, and angiogenic properties. Introduction of EVs helped mitigate damage from reperfusion (Chen et al., 2021). EVs have been utilized as a therapeutic approach in immune-mediated illness, in addition to their application in intentionally induced hypoxia to organs as in transplantation or cardiac surgery. In mice, EVs generated from IDO-positive DCs successfully delayed the onset of collagen-induced arthritis (Bianco et al., 2009). IDO expression in dendritic-cell-derived EVs that were driven into host cells is considered to have reduced this impact.

The application of such findings *in vivo* remains a distant dream. However, deriving EVs from IDO-expressing DCs may be a therapeutic target, particularly in an immune-compromised state characterized by such variable facets as SIRS (Raeven et al., 2018). EVs may eventually be used as biomarkers in sepsis, as they contain critical nucleic acids involved in the suppression of inflammatory responses (Raeven et al., 2018). An in-depth examination of the nucleic acid profiles of EVs, with a focus on immune-modulatory enzymes such as IDO, could be used to assess the inflammatory state.

Knowing that oxidative stress plays an important role in surgical trauma response, these findings point to IDO’s potentially therapeutic and prognostic function in addressing the surgical stress cascade. Apart from sepsis, whether the chemicals described above offer a treatment alternative for the oxidative stress-induced inflammatory response by surgical trauma remains unknown, since the complicated role of IDO is currently being investigated. However, surgical trauma response shares several pathways with SIRS, sepsis, and anoxic states. The final common pathway of a vicious circle ending in cell death by necrosis or apoptosis, in which IDO plays an important role, could potentially be altered by targeting IDO early enough. By altering the oxidative damage by surgical trauma, surgical outcome could be significantly improved.

## Future Perspectives

In patients undergoing surgery, all sub-specialties of a multidisciplinary team must be involved in order to optimize patient outcomes. Anesthesia has a vital role in decreasing patient stress in the pre- and perioperative settings. On anesthesia induction, strong opioids such as morphine or high-dose fentanyl suppress the postsurgical stress response. However, they are limited by serious ventilatory problems at higher doses (Hall, 1985; Abd Elrazek et al., 1999). Clonidine, a potent alpha-2 agonist, can also positively affect postsurgical sympathetic tone, reducing the stress response (Aantaa and Scheinin, 1993). Regional anesthesia, such as epidural blocking T4-S5, has a positive effect on post-surgical cortisol levels (Engquist et al., 1977). Thoracic epidurals even lower the troponin T (TnT) values in patients undergoing CPB (Loick et al., 1999). However, this effect is limited by frequently concomitant anticoagulant therapy, increasing the risk of epidural hematomas and impaired neurologic outcomes.

**FIGURE 5:**
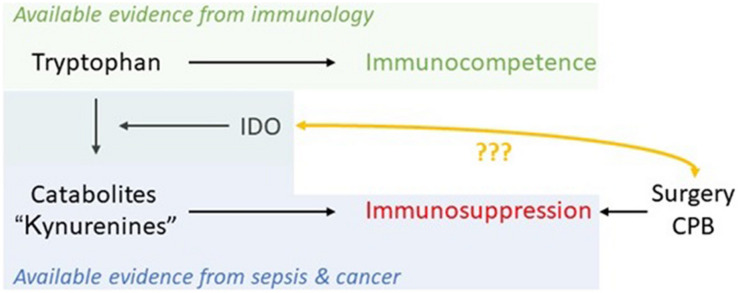
Schematic of available evidence from different fields and the potential of IDO as a marker in the perioperative period to monitor immunosuppression caused by major trauma/physiological derangement such as cardiopulmonary bypass (CPB) in cardiac surgery.

Indoleamine-2,3-dioxygenase 1 activation and emptying of Trp levels has been found to be stimulated *via* cytokine-induction by psychological stress also (Raeven et al., 2018). Thus, anesthetists play a basic role in limiting surgical stress. As there are sufficient data demonstrating increased Trp metabolism already prior to surgery, it should be possible in the near future to use IDO as a biomarker in patient-centered decision-making, providing optimal treatment in the perioperative period (Figure 5; Kiank et al., 2010).

A number of factors, including the fact that most research is conducted on mouse models, complicate the use of IDO as a biomarker and therapy target at the current stage. Especially when it comes to studying myeloid cells in mice, these models might not be representative (Schmidt and Schultze, 2014), as mice seem not to express IDO in myeloid cells (Blumenthal et al., 2012). Also, measuring IDO is laborious. Using Trp levels as a surrogate for IDO activity is not helpful, since Trp can be metabolized either by IDO1, IDO2, or TDO in the Kyn direction or *via* the serotonin pathway. This limits our ability to draw direct conclusions about IDO activity. Patients with sepsis or SIRS in clinical practice usually show significant variability in factors such as age, presence of underlying or secondary illness, immune system status, and infection severity. There are several pathophysiological processes that can result from an infection, and the name “sepsis” encompasses them all. These characteristics of heterogeneity make it more difficult to detect sepsis quickly and to select the best therapy for each patient. To make therapy decisions and prognosis decisions in infections, a metabolomic approach is preferable. Despite the fact that sepsis metabolism has been studied in the past, a multivariate score utilizing serum amino acid profiling as a sepsis diagnostic biomarker is yet to be developed. Despite several investigations into metabolomics in sepsis, only a small number have looked at serum amino acids as potential biomarkers. A recent study by Ahn et al. (2021) intended to design and verify a sepsis biomarker as a multivariate index based on amino acid profiling. Patients with SIRS were also included, a disease that can be difficult to identify from sepsis when pre-existing sepsis indicators are used (Ahn et al., 2021). The study might help clinicians and researchers better understand how metabolomics can be used in clinical medicine and how multivariate index analysis can be used to generate multivariate biomarkers that function well. More research, particularly with human probes, is urgently required for further understanding IDO in the process as a perioperative marker. Our group has initiated a biobank-derived study on cardiac surgical patients to analyze the impact of IDO as a perioperative marker of the immune system.

## Conclusion

Indoleamine-2,3-dioxygenase’s immuno-modulatory function alters the physiologically balanced immune state and has a role in disease etiology as well as perioperative settings after surgery. The role of IDO in autoimmune diseases, malignancies including glioblastoma, and organ transplantation has been widely investigated. But with severe systemic infections like sepsis, it is still not clear. IDO might offer validity as a prognostic biomarker and therapeutic target for immune-mediated illnesses. Finally, IDO offers potential as a pre-operative biomarker for all disciplines involved in the decision-making process and care of patients undergoing surgery.

## Author Contributions

CB, PH, MM, TC, and ML searched the literature and wrote the manuscript. All authors contributed to the article and approved the submitted version.

## Conflict of Interest

The authors declare that the research was conducted in the absence of any commercial or financial relationships that could be construed as a potential conflict of interest.

## Publisher’s Note

All claims expressed in this article are solely those of the authors and do not necessarily represent those of their affiliated organizations, or those of the publisher, the editors and the reviewers. Any product that may be evaluated in this article, or claim that may be made by its manufacturer, is not guaranteed or endorsed by the publisher.

## Abbreviations

AhR: aryl hydrocarbon receptor
BLIMP-1: B lymphocyte-induced maturation protein-1
CPB: cardiopulmonary bypass
CD: cluster of differentiation
COX: cyclooxygenase
CRP: C-reactive protein
CTLA-4: cytotoxic T-lymphocyte -associated protein
DAMP: damage-associated molecular pattern
DC: dendritic cell
EV: extracellular vesicle
GCN2: general control non-depressible 2
IDO: indoleamine-2,3-deoxygenase
IFN: interferon
IL: interleukin
ITIM: immune tyrosine-based inhibitory motif
Kyn: kynurenine
LAG3: lymphocyte-activation gene 3
MHC: major histocompatibility complex
mTOR: mechanistic target of rapamycin (previously: mammalian target of rapamycin)
MS: multiple sclerosis
NAD: nicotinamide adenine dinucleotide
NF- κ B: nuclear factor kappa-light-chain-enhancer of activated B cells
PAMPs: pathogen-associated molecular patterns
PD-L1: programmed death-ligand 1
PD-1: programmed cell death protein 1
PGE2: prostaglandin E2
PRR: pattern recognition receptor
RNA: ribonucleic acid
SHP: Src homology 2 domain phosphatase
SIRS: severe inflammatory response syndrome
STAT: signal transducer and activator of transcription protein
TDO: tryptophan-2,3-dioxygenase
Teff: effector T-cell
TGF- β: transforming growth factor beta
T_*h*_: T helper cell
TNF- α: tumor necrosis factor alpha
TnT: troponin T
Tregs: T regulatory cells
Trp: tryptophan.
